# Peroxisomal Membrane Contact Sites in Mammalian Cells

**DOI:** 10.3389/fcell.2020.00512

**Published:** 2020-06-23

**Authors:** Chao Chen, Jing Li, Xuhui Qin, Wei Wang

**Affiliations:** ^1^Department of Orthopaedics, Union Hospital, Tongji Medical College, Huazhong University of Science and Technology, Wuhan, China; ^2^Department of Integrated Traditional Chinese and Western Medicine, Tongji Hospital, Tongji Medical College, Huazhong University of Science and Technology, Wuhan, China; ^3^Department of Human Anatomy, School of Basic Medicine, Tongji Medical College, Huazhong University of Science and Technology, Wuhan, China

**Keywords:** peroxisomes, membrane contact sites, tethering complexes, metabolism, organelle crosstalk

## Abstract

Peroxisomes participate in essential cellular metabolic processes, such as oxidation of fatty acids (FAs) and maintenance of reactive oxygen species (ROS) homeostasis. Peroxisomes must communicate with surrounding organelles to exchange information and metabolites. The formation of membrane contact sites (MCSs), where protein-protein or protein-lipid complexes tether the opposing membranes of two organelles, represents an essential means of organelle crosstalk. Peroxisomal MCS (PO-MCS) studies are emerging but are still in the early stages. In this review, we summarize the identified PO-MCSs with the ER, mitochondria, lipid droplets, and lysosomes in mammalian cells and discuss their tethering mechanisms and physiological roles. We also highlight several features of PO-MCSs that may help future studies.

Peroxisomes are essential single-membrane-bound organelles present in virtually all eukaryotic cells (Smith and Aitchison, 2013). Peroxisomes play indispensable roles in both catabolic (degradative) and anabolic (biosynthetic) metabolism through the enzymes located in the peroxisomal matrix (Waterham et al., 2016). The primary catabolic pathways include the oxidation of fatty acids (FAs) and the detoxification of glyoxylates. The main anabolic pathways include the biosynthesis of bile acids, ether phospholipids, and docosahexaenoic acids. In addition, peroxisomes are involved in the production and decomposition of reactive oxygen species (ROS), which is partly coupled to the above peroxisomal metabolic pathways. Peroxisomes can serve as signaling platforms for antiviral innate immunity (Cook et al., 2019), and ROS modulated mTORC1 activity (Zhang et al., 2013, 2015).

Peroxisome deficiency causes severe human diseases, emphasizing the crucial role of peroxisomes. The diseases fall into two main categories: single peroxisomal enzyme deficiencies and peroxisome biogenesis disorders (PBDs) (Waterham et al., 2016). PBDs are inherited in an autosomal recessive manner and affect the overall assembly and function of peroxisomes. Patients with Zellweger syndrome (ZS), the most severe form of PBDs, fail to make any developmental progress and die at an early age (Suzuki et al., 2001). Depending on the organism, tissue, cell type, and environmental factors, the functions of peroxisomes can change to meet the metabolic needs of cells (Mast et al., 2020).

Organelles are cellular compartments that perform specific enzymatic reactions. However, organelles cannot exert their activities alone and must exchange information and metabolites with others to coordinate cellular functions. Several mechanisms have been proposed for this crosstalk, including vesicular trafficking and signal transduction pathways (Shai et al., 2016). Accumulating studies now show that membrane contact sites (MCSs) represent a fast and efficient way to communicate between organelles (Prinz et al., 2020). A bona fide MCS is defined as follows: (1) close apposition (10∼30 nm) of the membranes from two tethered organelles; (2) lack of membrane fusion or transient hemifusion may occur; (3) enrichment of specific proteins and/or lipids at the MCS; and (4) regulation of the composition and/or function of one or two of the organelles (Prinz, 2014).

For many years, electron microscopy (EM) studies in fungi, plants, and mammals revealed that peroxisomal membranes are juxtaposed to many other organelles, such as the ER, lipid droplets (LDs), plasma membrane (PM), mitochondria, and chloroplasts (Schrader et al., 2013). The simultaneous fluorescent labeling of six organelles in COS-7 cells reveals that peroxisomes associate with the ER, mitochondria, LDs, the Golgi, and lysosomes (Valm et al., 2017). To date, the tethering complexes and physiological roles of peroxisomal MCSs (PO-MCSs) with the ER, mitochondria, LDs, and lysosomes have been investigated in mammalian cells (Castro et al., 2018; Sargsyan and Thoms, 2020; Schrader et al., 2020). In this review, we focus on these identified PO-MCSs in mammalian cells and discuss several features of PO-MCSs.

## Peroxisome-ER-MCSs

In human cells, the biosynthesis of unsaturated FAs, sterols, ether phospholipids, and bile acids involves both the ER and peroxisomes and requires intimate crosstalk. Metabolic defects in these organelles lead to severe human diseases (Schrader et al., 2013). The ER represents the largest membrane-bound organelle in eukaryotic cells and forms MCSs with many other organelles, including peroxisomes. Although peroxisome-ER (PO-ER) associations were identified by EM in mammalian cells nearly 50 years ago (Novikoff and Novikoff, 1972), the MCSs mediating their crosstalk remained elusive.

**FIGURE 1:**
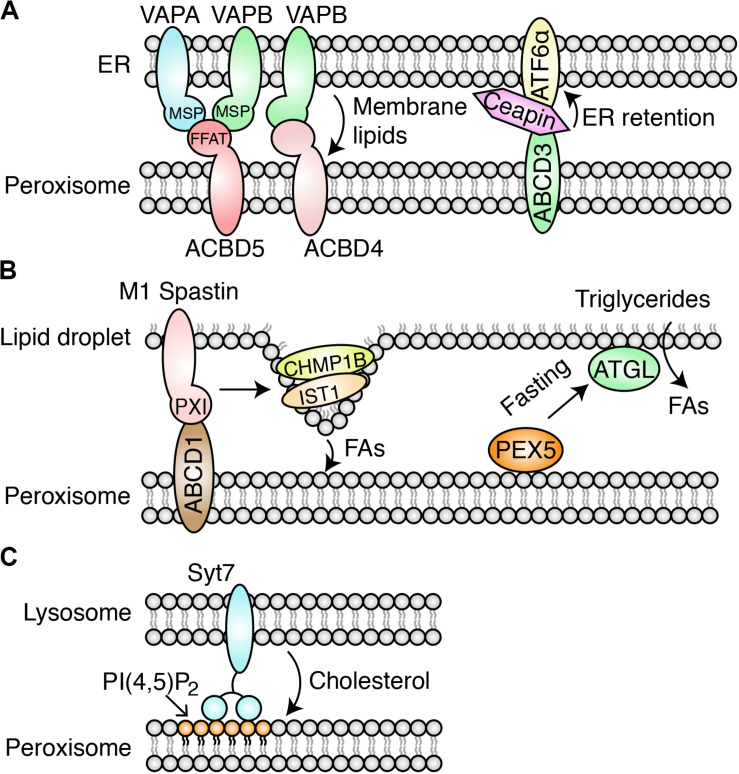
Identified PO-MCSs in mammalian cells. **(A)** PO-ER-MCS. At the MCS, the FFAT motif in ACBD5 interacts with the MSP domain in VAPA/B. The ACBD4/5-VAPA/B-tethered MCS facilitates lipid transfer from the ER to peroxisomes and promotes the expansion of peroxisomal membranes. Ceapin sequesters ATF6α at the ER by tethering ATF6α to peroxisomal ABCD3. **(B)** PO-LD-MCS. M1 Spastin interacts with ABCD1 through the peroxisome-interacting (PXI) region. M1 Spastin recruits the ESCRT-III subunits IST1 and CHMP1B to the surface of LDs to promote FA trafficking through the MCS. ATGL is recruited to the MCS by PEX5 in response to fasting stress, promoting lipolysis of triglycerides stored in LDs. **(C)** PO-Lyso-MCS mediated by PI(4,5)P_2_ and Syt7. This MCS facilitates cholesterol trafficking from lysosomes to peroxisomes.

Acyl-CoA binding domain containing 5 (ACBD5) is a peroxisomal tail-anchored membrane protein, and its deficiency can cause a defect in peroxisomal very-long-chain FA metabolism (Ferdinandusse et al., 2017). ACBD5 and its fungal ortholog, ATG37, have been suggested to play critical roles in phagophore formation during pexophagy (Nazarko et al., 2014). VAPA and VAPB (VAPA/B) are tail-anchored and ER vesicle-associated membrane proteins. These proteins can act as the MCS tether through the major sperm protein (MSP) domain that interacts with the two phenylalanines in an acidic tract (FFAT) motif of client proteins localized in the opposing organelles (Lev et al., 2008). Two independent studies showed that the tether ACBD5-VAPA/B mediates the formation of PO-ER-MCS (Costello et al., 2017a; Hua et al., 2017; Figure 1A).

In these studies, super-resolution microscopy and EM visualization revealed that the two organelles were in proximity to each other. Cooverexpression of ACBD5 and VAPA/B or their knockdowns increases and decreases MCS formation, respectively, suggesting that tethering relies on the ACBD5-VAPA/B interaction. As shown for other VAPA/B interacting proteins, ACBD5 interacts with the MSP domain of VAPA/B through the FFAT motif. ACBD4, a second ACBD family protein, also interacts with VAPB for PO-ER associations (Costello et al., 2017b; Figure 1A).

Peroxisomes can proliferate through the growth and division model, which involves membrane elongation, constriction, and fission (Schrader et al., 2012). Since peroxisomes share division proteins with mitochondria (e.g., DRP1, MFF, and FIS1) (Smith and Aitchison, 2013) and ER-MCSs have been shown to mark mitochondrial or endosomal fission sites and modulate their division (Friedman et al., 2011; Rowland et al., 2014), it is reasonable to hypothesize that PO-ER-MCSs play a role in peroxisome division. However, peroxisome biogenesis is unaffected in ACBD5-deficient cells (Ferdinandusse et al., 2017; Yagita et al., 2017), and peroxisome morphology is normal in cells with reduced PO-ER-MCSs due to ACBD5/VAP silencing (Costello et al., 2017a; Hua et al., 2017). These results suggest that the ACBD5-VAPA/B tethered MCSs may not play a role in peroxisome division.

The phospholipid contents of peroxisome membranes are rich in phosphatidylcholine (PC) and phosphatidylethanolamine (PE), similar to the contents of the ER (Hardeman et al., 1990). The peroxisome is unable to generate membrane lipids locally because it does not contain biosynthesis enzymes. MFF and DRP1 are two crucial molecules involved in peroxisome and mitochondrion fission, and their deficiencies result in pronounced elongation of the two organelles, likely through a constant transfer of lipids from the ER to peroxisomes (Schrader et al., 2012). It has been found that disrupting the ACBD5-VAPA/B tethered MCS in MFF- or DRP1-deficient cells significantly inhibited the elongation of peroxisomes but not of mitochondria, suggesting that the MCS may supply the lipids required for peroxisome membrane expansion.

Both groups found that disrupting the ACBD5-VAPA/B tethered MCS increases the movement of peroxisomes, suggesting that the MCS negatively regulates peroxisome motility through ER anchoring. A motility-restricting role is also indicated for the yeast PO-ER-MCS, which can retain a fraction of peroxisomes by anchoring with the ER in mother cells when peroxisomes passage into the daughter cells (Knoblach et al., 2013). In addition, Hua et al. (2017) found a potential role of PO-ER-MCSs in the synthesis of plasmalogen phospholipids and the maintenance of cellular cholesterol levels. VAP proteins also play roles in lipid transfer at ER-MCSs with other organelles, such as the Golgi, mitochondria, and LDs (Kamemura and Chihara, 2019). Expression of an artificial PO-ER tether without any functional domain partially restored peroxisomal membrane expansion after ACBD5 silencing (Costello et al., 2017a), suggesting that VAPA/B and ACBD5 do not play an active lipid-transferring role in PO-ER-MCSs. Deficiency in both the ACBD5 and VAPB genes has been implicated in human disorders, emphasizing the significance of the identified PO-ER-MCSs (Kim et al., 2010; Abu-Safieh et al., 2013; Ferdinandusse et al., 2017; Yagita et al., 2017).

Other PO-ER-MCSs have also been identified. The MCS tethered by the peroxisomal PI(4,5)P2 and ER-resident extended synaptotagmins (E-Syts) may facilitate the transport of cholesterol from the peroxisome to the downstream ER organelle (Xiao et al., 2019). ATF6α is an unfolded protein response (UPR) sensor, which traffics to the Golgi apparatus for proteolysis and its subsequent movement to the nucleus for transcriptional activation during ER stress (Haze et al., 1999). A genome-wide CRISPR interference screening revealed that the drug Ceapin induces PO-ER-MCS formation by forcing the interaction between ATF6α and the peroxisomal transmembrane protein ABCD3 (Torres et al., 2019). The ATF6α-ABCD3 interaction sequesters ATF6α at the ER and inhibits its activity as the UPR sensor (Figure 1A). Notably, the MCS does not require the known ACBD4/5-VAPA/B tether. The study suggests that Ceapin may be used for the treatment of cancer, since cancer development relies on active ATF6α signaling; this finding presents a novel drug-development strategy for targeting the MCS.

## Peroxisome-Mitochondrion-MCSs

Peroxisomes and mitochondria must communicate with each other to meet the metabolic needs of cells. The best examples are illustrated by the β-oxidation of FAs and metabolism of ROS (discussed below). These processes share some crucial division proteins (Delille et al., 2009), de novo biogenesis of peroxisomes relies on mitochondrion-derived vesicles (Sugiura et al., 2017), and these organelles function cooperatively in antiviral signaling and defense (Kagan, 2012). In addition, defective mitochondria are observed in several peroxisomal disorders (Baumgart et al., 2001; Lopez-Erauskin et al., 2013).

In the 1970s, a close spatial peroxisome-mitochondrion (PO-Mito) association was observed in the myocardium of rodents and primates by a peroxidative catalase activity-based alkaline DAB staining method (Hicks and Fahimi, 1977). Overexpression of PEX11β, a critical peroxisome division factor, induces the formation of mitochondrion-interacting membrane protrusions (Kustatscher et al., 2019). The PO-Mito interaction increases when cells are infected with an RNA virus, suggesting a critical role of PO-Mito-MCSs in RNA virus infections (Horner et al., 2011). The role of PO-Mito-MCSs has been implicated in the transfer of metabolites involved in steroid biosynthesis in mouse Leydig tumor cells (Fan et al., 2016). This study suggests that the tethering complex consists of a splice isoform of an acyl-CoA binding domain-containing protein, enoyl-CoA δ isomerase 2 (ACBD2/ECI2). However, as ACBD2/ECI2 proteins are transported into the matrix of both mitochondria and peroxisomes, their tethering role needs further investigation.

Both peroxisomes and mitochondria possess separate β-oxidation pathways in mammalian cells, which are different from those in yeast and plants, whose peroxisomes are the sole organelles for β-oxidation of FAs. The enzymatic steps of β-oxidation of FAs in these two organelles are similar but differ in the catalyzed substrates. For example, peroxisomes preferentially catalyze the β-oxidation of very-long-chain FAs, while the mitochondrial β-oxidation system catalyzes medium and long-chain FAs. The chain-shortened FAs, as well as the acetyl-CoA generated through peroxisomal β-oxidation, need to be directed to mitochondria for further oxidation and ATP production. Different mechanisms have been proposed for the transfer of these metabolites, including the carnitine system, membrane pores, and vesicular transport (Antonenkov and Hiltunen, 2012; Sugiura et al., 2014). A high content screening approach for PO-MCSs in yeast suggests that the tethering complex (Pex11, Fzo1, and Pex34) contributes to PO-Mito-MCS formation, which facilitates the transfer of β-oxidation products (Shai et al., 2018). The conserved function of mammalian PO-Mito-MCS remains to be determined.

In addition, both organelles contain pro-oxidant and antioxidant systems. Increasing evidence demonstrates that these organelles undergo intimate crosstalk during cellular redox metabolism (Fransen and Lismont, 2018). For example, inhibition of the peroxisomal antioxidant enzyme catalase or excess ROS generation in peroxisomes disrupts mitochondrial redox (Walton and Pizzitelli, 2012; Wang et al., 2013). Expression of peroxisome-targeted PRDX5 (Peroxiredoxin-5) protects cells from the oxidative insults generated from mitochondria (Walbrecq et al., 2015). The ER-Mito-MCS has been shown to transfer ER-derived ROS to mitochondria, causing the cell to be sensitive to mitochondrial apoptosis (Verfaillie et al., 2012). ER-Mito-MCS-mediated Ca^2+^ transfer stimulates ROS translocation from mitochondria to the MCS. The local H_2_O_2_ enrichment at the MCS further augments the ER-Mito Ca2^+^ flux, likely through H_2_O_2_-mediated oxidation of specific thiol groups of the pump receptor IP3 (Booth et al., 2016). To date, no studies have shown the role of PO-Mito-MCS in redox crosstalk. It is tempting to speculate that such a PO-Mito-MCS exists for maintaining cellular redox balance.

The above results suggest that MCSs play critical roles in PO-Mito crosstalk. However, the mechanism of PO-Mito-MCSs remains elusive. Future studies identifying the tethering components would increase our understanding of PO-Mito crosstalk.

## Peroxisome-Lipid Droplet-MCSs

Lipid droplets are essential and significant lipid storage organelles for neutral lipids, such as triacylglycerol and cholesterol ester. The lipids must be imported into oxidative organelles, such as mitochondria and peroxisomes, for β-oxidation to enable cellular homeostasis (Thiam and Dugail, 2019). Indeed, aberrant FA metabolism in LDs is implicated in severe physiological disorders, including lipodystrophy and neurological diseases (Welte, 2015). Defective FA metabolism in peroxisomes causes an accumulation of LDs, as found in patients with peroxisomal disorders (Schaumburg et al., 1972; Engelen et al., 2012). Changes in the number and size of LDs are observed in peroxisome-deficient mice (Baes et al., 1997; Dirkx et al., 2005). These results suggest that the two organelles need to cooperate to maintain cellular homeostasis. Two recent studies find that they communicate through the peroxisome-lipid droplet (PO-LD)-MCSs as follows (Chang et al., 2019; Kong et al., 2020).

Spastin is a microtubule-severing protein that belongs to the AAA (ATPases Associated with various cellular Activities) family. The longer M1 variant (M1 Spastin) encodes an N-terminal hydrophobic hairpin motif, which targets M1 Spastin to LDs (Errico et al., 2002). Chang et al. (2019) demonstrated that M1 Spastin forms a tethering complex with the peroxisome membrane protein ABCD1 to maintain the PO-LD MCS for FA trafficking from LDs to peroxisomes. M1 Spastin also recruits the curvature-generating ESCRT III subunits IST1 and CHMP1B to the surface of LDs to promote FA trafficking (Figure 1B). The trafficking of FAs through the MCS prevents the accumulation of peroxidated lipids following oxidative stress (Chang et al., 2019). Spastin gene mutations are the most common cause of hereditary spastic paraplegias (HSPs), a group of inherited neurological disorders (Blackstone, 2018). The results suggest that failure to induce LD-PO-MCS formation and subsequent FA transport by the M1 Spastin mutation (K388R) may contribute to HSP (Chang et al., 2019), highlighting the critical role of PO-LD-MCS in HSP pathogenesis.

Another study revealed the role of PO-LD-MCS in regulating lipolysis, the hydrolysis of lipid metabolites stored in LDs (Kong et al., 2020). During fasting, the increase in PO-LD-MCSs facilitates the spatial translocation of adipose triglyceride lipase (ATGL) onto LDs for lipolysis. PEX5 recruits ATGL to the MCS, independent of its role as the receptor for the import of peroxisomal matrix proteins (Figure 1B). It would be interesting to investigate whether the M1 Spastin-ABCD1 complex tethers this MCS for lipolysis. Importantly, these results are verified by studies in multiple models, such as mammalian adipocytes, *Caenorhabditis elegans*, and mice. The study reveals the physiological significance of the PO-LD-MCS in maintaining energy homeostasis in response to nutritional status. As aberrant lipolysis is associated with severe metabolic diseases, such as obesity and diabetes, this study suggests PO-LD-MCSs as a potential therapeutic target for these diseases.

As discussed above, the MCS can facilitate the transport of lipids from LDs to peroxisomes for their breakdown or function as a station enriched with lipases to hydrolyze the lipids in LDs. Detection of peroxisome-derived metabolites (e.g., ether-linked lipids) in LDs suggests that they may be transferred from peroxisomes to LDs through the MCS (Bartz et al., 2007). The bidirectional transfer at the PO-LD-MCS awaits discovery.

## Peroxisome-Lysosome-MCSs

Lysosomes are degradative and metabolic organelles. Many essential metabolic processes, such as those for cholesterol, occur in this organelle. Cholesterol is a crucial component for maintaining the fluidity, permeability, and organization of mammalian membranes. Cholesterol is unevenly distributed in cellular membrane structures, with the highest contents (60∼80% of total cellular cholesterol) in the PM (Lange et al., 1989). The low-density lipoprotein-derived cholesteryl ester is internalized through endocytosis, hydrolyzed to unesterified cholesterol in lysosomes and further delivered to downstream organelles (Chang et al., 2006).

A genome-wide pooled shRNA screen identified a list of peroxisome genes required for cholesterol trafficking (Chu et al., 2015). These genes are either essential for peroxisome biogenesis or involved in its metabolism. These authors observed a dramatic accumulation of cholesterol in lysosomes and a significant reduction of peroxisome-lysosome (PO-Lyso)-MCS formation in cells deficient in these peroxisome genes. These authors further revealed that peroxisomal PI(4,5)P_2_ lipid and lysosomal synaptotagmin VII (Syt7) protein tether the MCS for cholesterol trafficking from lysosome to peroxisome (Chu et al., 2015; Figure 1C). Time-lapse visualization reveals that the PO-Lyso-MCS is transient. Depletion and repletion of cholesterol inhibit and recover the MCS, respectively, indicating that MCS formation depends on the presence of cholesterol. The follow-up study found that PIP4K2A, a PI(5)P-kinase, contributes to the generation of peroxisomal PI(4,5)P2, enhances PO-Lyso-MCS formation, and promotes cholesterol trafficking (Hu et al., 2018).

Drastic amounts of cholesterol accumulate in the mouse model and the patient fibroblasts with peroxisomal disorders, as these authors have examined. This study suggests that part of the pathological mechanisms of peroxisomal disorders may be attributed to the blockage of cholesterol trafficking caused by defective PO-Lyso-MCS formation and may provide novel strategies for their diagnoses and treatments (Chu et al., 2015). However, as the experimental method for peroxisome purification in this study is under debate (Schrader et al., 2020) and the study does not examine the loss of PO-Lyso-MCS in these patient fibroblasts, other mechanisms may contribute to abnormal cholesterol accumulation. Studies have shown that the initial step of plasmalogen synthesis occurs in peroxisomes and that its deficiency interferes with the transport of cholesterol from the PM or endocytic compartments to the ER (Thai et al., 2001; Munn et al., 2003). The accumulation of lysosomal cholesterol in peroxisome-deficient cells could result from insufficient plasmalogen synthesis due to loss of peroxisome functions.

In addition, lysosomes can serve as the signaling hub for the vital growth regulator mTOR. When there are sufficient nutrients, mTOR is recruited to lysosomes and activated by lysosomal Rheb proteins to promote downstream anabolism for cellular growth (Saxton and Sabatini, 2017). Interestingly, a study showed that peroxisome-localized TSC1/2 proteins function as Rheb GTPase-activating proteins to repress mTOR signaling in response to the ROS generated within peroxisomes (Zhang et al., 2013). It is unknown how mTOR signaling is coordinated in response to nutrients and oxidative stresses. The PO-Lyso-MCS enriched with Rheb proteins may fulfill such a role, similar to that proposed for the lysosome-Golgi-MCS in the activation of mTOR signaling (Hao et al., 2018). However, TSC1/2 proteins were not detected in several proteomic studies of purified peroxisomes (Yifrach et al., 2018), and they dissociate from the lysosomes to the cytosol upon insulin stimulation, resulting in Rheb-mediated mTORC1 activation at lysosomes (Menon et al., 2014). These findings do not support the role of PO-Lyso-MCS in modulating mTORC1 signaling, as hypothesized above.

## PO-MCSs With Other Organelles

In addition, PO-MCSs with other organelles have been indicated. Peroxisomes move to the cell periphery near the PM by ACBD5 overexpression in neurons. This peroxisome redistribution does not depend on the interaction of ACBD5 with VAPs, suggesting that other ACBD5 interacting proteins may tether peroxisomes to PM (Wang et al., 2018). Live imaging of fusion cells expressing peroxisomal proteins fused to different fluorescence tags reveals that peroxisomes can interact with each other in a transient and long-term manner. The contact does not promote the exchange of matrix and membrane proteins, FAs, and H_2_O_2_ (Bonekamp et al., 2012). Global analysis of the organelle interactome reveals that peroxisomes contact the Golgi, in addition to the ER, mitochondria, LDs, and lysosomes (Valm et al., 2017). With more PO-contacting organelles being identified, their tethering components and functions remain to be characterized.

## Discussion

The MCSs open an avenue for peroxisomes to communicate with other organelles for diverse purposes, including organelle positioning, lipid supply for membrane elongation, and metabolism coordination, as discussed above. However, MCSs are much more complicated than initially thought. We highlight and discuss several features of PO-MCSs that may help future studies.

### Heterogeneity of Peroxisomes

Peroxisomes are heterogeneous in terms of their number, morphology, distribution, and composition in different cells or environmental settings (Mast et al., 2020). For example, the number of peroxisomes increases when rodents are administered fibrate derivatives but decreases rapidly upon withdrawal of the drugs (Fahimi et al., 1982; Yokota, 1986; Yokota, 1993). Enveloped virus infection induces significant peroxisome biogenesis, promoting phospholipid plasmalogen synthesis for virus production (Jean Beltran et al., 2018). Peroxisomes also display heterogeneity in the same cell. An example is that two populations of peroxisomes that contain different ratios of lipid β-oxidation enzymes to catalase are isolated in HepG2 cells (Schrader et al., 1994). The population containing higher contents of β-oxidation enzymes may favor PO-MCSs for FA metabolism. In contrast, those with higher catalases may prefer MCSs for ROS crosstalk. Hence, PO-MCSs may differ in terms of their tethering mechanisms and functions under these varying conditions, and selecting an appropriate cell or animal model would facilitate PO-MCS studies.

### Unconventional PO-MCSs

The identified PO-MCSs are tethered through different protein-protein or protein-lipid complexes and manifest a variety of physiological roles. In addition, as each cellular organelle has a unique structure, the mode of PO-MCS formation may be organelle-specific, as discussed below.

Mitochondria are surrounded by double membranes, which separate the organelle into two compartments, the intermembrane space and the matrix. Peroxisomes may contact the outer membranes, as shown for the ER-Mito MCS (Murley and Nunnari, 2016). This MCS may indirectly facilitate metabolic exchange with the matrix through transporters located at the inner membranes. Alternatively, it is tempting to speculate that the PO-tethering complexes may extend into the inner membranes to facilitate the exchange of metabolites.

Compared to bilayer-bound organelles, LDs have phospholipid monolayer membranes that surround the lipid core. This unique monolayer of LDs may be continuous with the outer leaflet of the bilayer of apposing organelles, as has been shown for ER-LD-MCS (Wang et al., 2016). This MCS does not exist between bilayer membranes and is distinct from the traditional MCS. In yeast, PO-LD-MCS formation increases in peroxisome-inducing conditions. The MCSs are enriched in β-oxidation enzymes, suggesting their roles in FA transfer from LDs to peroxisomes. At these MCSs, peroxisomal protrusions extend into the core of LDs, likely representing the “bridging contact” (Binns et al., 2006). It remains to be investigated whether peroxisomes form such a “bridging contact” with LDs in mammalian cells.

### The Components at the PO-MCSs

The identified PO-MCSs are tethered through the protein-protein or protein-lipid complexes present on the opposing membranes of the organelles. The tethering complexes may play additional roles in the MCSs. For example, the tether M1 Spastin also recruits membrane curvature-shaping proteins to promote lipid transfer at the PO-LD-MCS (Chang et al., 2019). The identified peroxisomal tethers play MCS-independent roles, such as in metabolic/enzymatic activities. Hence, to elucidate whether the phenotype is solely caused by defective PO-MCS formation, it is necessary to test if a mutant defective in its tethering activity but maintaining other activities inhibits PO-MCS functions. Multiple tethering pairs may also exist for the PO-MCS, as shown for the PO-ER-MCSs as discussed above and for other organelle MCS (Scorrano et al., 2019). In this scenario, loss of the identified PO-MCS tether does not necessarily reduce their contacts and/or functions because of the redundancy of the tethering complexes. In addition, other effector proteins may be enriched at the MCSs to regulate MCS formation or facilitate the exchange of metabolites (Scorrano et al., 2019). As discussed above, ATGL can be grouped as the effector protein that promotes lipolysis at the PO-LD-MCS during fasting (Kong et al., 2020).

Although some components of the PO-MCS have been elucidated as discussed above, many questions remain to be answered. Elucidating components, such as tethering complexes and effector proteins, at PO-MCSs is a major challenge but will profoundly increase our understanding of PO-MCSs.

### Coordination of PO-MCSs With Multiple Organelles

Membrane contact sites do not function alone. MCSs must be integrated and coordinated in response to the changing cellular environment. Peroxisomes can contact multiple organelles simultaneously, as shown for the PO-interacting network in COS-7 cells (Valm et al., 2017). The peroxisome-contacting organelles can be shifted to other organelles in varying conditions. An example is that PO-Mito contact increases when MAM (mitochondrial associated membrane, a specialized ER subdomain)-mitochondria contacts are disrupted, resulting in a concomitant increase in IFN-β signaling during virus infection (Horner et al., 2011). We discuss three potential mechanisms for coordinating PO-MCSs with multiple organelles as follows.

A study investigated the assembly of ER-MCSs with a variety of organelles through lipid-based phase separation (King et al., 2020). By employing hypotonic cell swelling, the ER and other membrane-bound organelles can be converted into micrometer-scale large intracellular vesicles (LICVs). Upon cooling, the ER-derived LICVs phase-partition into the ER ordered (ER_*o*_) and disordered (ER_*d*_) lipid domains. Interestingly, they find that the PO-ER-MCS is located at the ER_*o*_/ER_*d*_ interphase, involving association with a mitochondrion. A yeast study also found that peroxisomes contact the ER and mitochondria at the three-way junction through Pex11 interacting with Mdm34, a component of ERMES (ER-mitochondria encounter structure) (Mattiazzi Usaj et al., 2015). As the three organelles are crucial for lipid and redox metabolism, the MCS at the tri-junction interface may allow efficient transfer of these metabolites among them. Hence, establishing PO-MCSs at the junction interface may coordinate peroxisome crosstalk with multiple organelles to meet metabolic needs.

Membrane contact site coordination can be achieved through the regulation of common tethering molecules (Harper et al., 2020). For example, in yeast, the tethering proteins Lam6 and Vps13 proteins are present at multiple contact sites, and they shift from one MCS to the other in response to changing carbon sources (Elbaz-Alon et al., 2015; Lang et al., 2015). As discussed above, The ER-localized VAPA/B proteins act as tethers with many other organelles, including peroxisomes (Eisenberg-Bord et al., 2016) and the PI(4,5)P_2_ lipid at peroxisomal membranes tethers peroxisomes to both lysosomes and the ER. Regulation of these common PO tethers, such as VAPA/B and PI(4,5)P_2_, may provide mechanistic hints for PO-MCS coordination.

The movement of peroxisomes in mammalian cells preferentially depends on the microtubular network and allows peroxisomes to be uniformly dispersed for metabolism (Neuhaus et al., 2016). PO-LD-MCS depends on the motility of peroxisomes along microtubules through the kinesin-like motor KifC3 to promote lipolysis under fasting stress conditions (Kong et al., 2020). Microtubule disruption by nocodazole treatment in COS-7 cells decreases peroxisome contacts with all the examined organelles (the ER, mitochondria, LDs, Golgi, and lysosomes) (Valm et al., 2017). These studies suggest that organelle motility is critical for PO-MCS formation. In turn, the MCSs regulate the movement and position of peroxisomes, exemplified by the role of PO-ER-MCS in restricting peroxisome motility, as discussed above. Peroxisomes can hitchhike on the microtubule-based motor machinery of endosomes for long-range movements through PO-endosome-MCSs in the filamentous fungus *Aspergillus nidulans*. Given the crucial role of microtubules in organizing a variety of organelle contacts and the reciprocal relationships between organelle motility and contact (de Forges et al., 2012; Valm et al., 2017), it is tempting to speculate that modulating the microtubular network can serve as another strategy for PO-MCS coordination.

## Concluding Remarks

In summary, the vital importance of peroxisomes is underscored not only by the essential metabolism within this organelle but also by the intimate crosstalk with other organelles. MCS formation is an efficient means by which peroxisomes communicate with other organelles. Some of the tethering mechanisms and physiological functions have been elucidated or suggested. These studies have increased our understanding of not only peroxisomes but also other organelles, including their biogenesis/turnover and metabolic functions. However, these studies are still in the early phase, and much that is unknown remains to be addressed. Future PO-MCS studies would shed novel insights into human diseases and determine their therapeutic targets.

## Author Contributions

CC, JL, and XQ wrote the draft of the manuscript. WW drew the figure and organized and proofread the manuscript. All authors contributed to the article and approved the submitted version.

## Conflict of Interest

The authors declare that the research was conducted in the absence of any commercial or financial relationships that could be construed as a potential conflict of interest.
